# Addition of TyG index to the GRACE score improves prediction of adverse cardiovascular outcomes in patients with non-ST-segment elevation acute coronary syndrome undergoing percutaneous coronary intervention: A retrospective study

**DOI:** 10.3389/fcvm.2022.957626

**Published:** 2022-08-25

**Authors:** Shuo Pang, Guangrui Miao, Yuanhang Zhou, Yang Du, Ziao Rui, Xiaoyan Zhao

**Affiliations:** Department of Cardiology, The First Affiliated Hospital of Zhengzhou University, Zhengzhou, China

**Keywords:** acute coronary syndrome, percutaneous coronary intervention, insulin resistance, biomarkers, prognosis

## Abstract

**Background:**

The Global Registry of Acute Coronary Events (GRACE) score is a widely recognized tool for predicting adverse cardiovascular events in patients with non-ST-segment elevation acute coronary syndrome (NSTE-ACS). The triglyceride-glucose index (TyG index) is a new biomarker of insulin resistance and has a close association with the occurrence of adverse cardiovascular events. We investigated whether the addition of the TyG index to the GRACE score could improve prognosis prediction in patients with NSTE-ACS undergoing percutaneous coronary intervention (PCI).

**Methods:**

In total, 515 patients with NSTE-ACS undergoing PCI were included in this retrospective study. Kaplan-Meier analysis was performed to describe the cumulative incidence of the primary endpoint based on the median TyG index. The relationship between the TyG index and GRACE score was analyzed using Spearman's rank correlation. Univariate and multivariate Cox proportional hazards analyses were used to identify independent risk factors. Based on the receiver operating characteristic curve, net reclassification improvement (NRI), integrated differentiation improvement (IDI), and decision curve analysis, the TyG index was evaluated for its predictive value when added to the GRACE score. ROC curve analyses, NRI, and IDI were used to compare the gain effect of the TyG index and the levels of HbA1C, FBG, TG, and LDL-C on the GRACE score for predicting adverse cardiovascular events.

**Results:**

The TyG index was an independent predictor of 2-year adverse cardiovascular events in patients with NSTE-ACS undergoing PCI. The addition of the TyG index to the GRACE score demonstrated an improved ability to predict 2-year adverse cardiovascular events compared with the GRACE score alone (AUCs: GRACE score 0.798 vs. GRACE score+TyG index 0.849, *P* = 0.043; NRI = 0.718, *P* < 0.001; IDI = 0.086, *P* < 0.001). The decision curve analysis suggested that the clinical net benefit of the new model (GRACE score+TyG index) was superior to that of the GRACE score alone, with a probability range of 0.04 to 0.32. When including the TyG index, HbA1C, FBG, TG, and LDL-C in the GRACE score system, we found that the TyG index had a greater incremental impact on risk prediction and stratification compared to the other parameters.

**Conclusion:**

Combining the TyG index and GRACE score could improve the prediction of 2-year adverse cardiovascular events. This new risk model could identify patients with NSTE-ACS at higher risk of adverse events following PCI so that they can be monitored more carefully.

## Introduction

Insulin resistance (IR) can induce disorders of glucose and lipid metabolism, which are important risk factors for the incidence and prognosis of cardiovascular diseases (1, 2). The hyperinsulinemia-glucose clamp test and homeostatic model assessment for insulin resistance (HOMA-IR) are recognized as accurate and reliable methods for evaluating IR; however, they are costly and complicated to perform. Epidemiological studies and clinical practice require easy-to-use markers of IR. The triglyceride-glucose index (TyG index) (composed of fasting glucose and triglycerides) not only has high sensitivity and specificity for IR identification but also correlates closely with the hyperinsulinemia-normal glucose clamp test and HOMA-IR and has been suggested to be a reliable alternative marker of IR (3). In addition, the TyG index has been proven to be closely associated with predicting the occurrence, progression, and prognosis of coronary heart disease (CHD) (4–6).

Even with percutaneous coronary intervention (PCI) and adequate antiplatelet drugs, the prognosis of some patients with non-ST-segment elevation acute coronary syndrome (NSTE-ACS) remains poor (7). Early risk stratification is important for clinical decision-making and prognostic assessment. The Global Registry of Acute Coronary Events (GRACE) risk score is currently the most widely used method for prognostic determination of patients with NSTE-ACS (8). Studies have shown that the GRACE risk score exhibits high predictive accuracy in both the inpatient phase and during long-term follow-up of major adverse cardiovascular events (MACEs) in patients with NSTE-ACS and guides clinicians in adjusting treatment regimens to reduce long-term cardiovascular risk (9). However, the TyG index, a new and powerful predictor of cardiovascular disease, has not been considered in the GRACE risk scoring system, which may result in a proportion of patients at high risk of adverse cardiovascular events being overlooked. Studies on the application of the TyG index in the GRACE score are limited; thus, the aim of this study was to investigate whether the predictive ability of the GRACE risk score improves when combined with the TyG index for 2-year adverse cardiovascular events in patients with NSTE-ACS and PCI.

## Materials and methods

### Study cohort

This was a single-center, retrospective study of patients with NSTE-ACS undergoing PCI at the First Affiliated Hospital of Zhengzhou University from April 2018 to December 2019, including those with unstable angina (UA) and non-ST-segment elevation myocardial infarction (NSTEMI).

The inclusion criteria were as follows: (1) diagnosis of UA or NSTEMI according to relevant guidelines (7); (2) PCI performed during hospitalization; and (3) integrated medical documentation. The exclusion criteria were as follows: (1) primary cardiomyopathy, valvular heart disease, and history of coronary artery bypass grafting; (2) severe liver or kidney dysfunction; (3) autoimmune diseases and hematologic disorders; (4) malignant diseases; and (5) use of triglyceride-lowering medications. Finally, a cohort of 515 patients with NSTE-ACS was included in the analysis (Supplementary Figure 1). This study was conducted in compliance with the Helsinki Declaration of Human Rights and was approved by the Clinical Research Ethics Committee of the First Affiliated Hospital of Zhengzhou University.

### Demographic and clinical data

Clinical data at admission, including demographic data, medical history, laboratory test results, and angiographic and echocardiographic data, were collected from the First Affiliated Hospital of Zhengzhou University Medical Information Recording System. Laboratory parameters were analyzed by standard techniques using fasting venous blood samples collected after overnight fasting (>8 h) before the baseline coronary procedure. Calculation of the GRACE score was based on the clinical data obtained at admission (age, heart rate, systolic blood pressure, Killip class, creatinine level, ST-segment deviation, elevated cardiac enzymes, and cardiac arrest) (Supplementary Table 1).

### Definitions

NSTE-ACS was defined according to the current guidelines of the European Society of Cardiology (10). A score of ≥140 was defined as high risk. Obesity was defined as a body mass index of >25 kg/m^2^. An estimated glomerular filtration rate <60 ml/min/1.73 m^2^ was considered to indicate the presence of chronic kidney disease. The TyG index was calculated as ln[fasting triglyceride (TG) (mg/dl) × fasting blood glucose (FBG) (mg/dl)/2].

### Outcomes and follow-up

The primary endpoint was defined as a composite of adverse cardiovascular events including all-cause death, non-fatal myocardial infarction, non-fatal ischemic stroke, and ischemia-driven revascularization. All-cause mortality was defined as death due to any reason. Reviews of electronic medical records and follow-up telephonic interviews were conducted to assess 2-year clinical outcomes.

### Statistical analyses

Continuous variables are expressed as mean ± standard deviation or median (interquartile range) according to the presence or absence of normal distribution. Comparisons between two groups were performed using Student's *t*-test or the Mann-Whitney U test. Categorical variables were expressed as numbers and percentages, and comparisons between groups were made using Pearson's chi-square test or Fisher's exact test. Kaplan–Meier analysis was performed to describe the cumulative incidence of the primary endpoint based on the median TyG index, and the groups were compared using the log-rank test. The correlation between the GRACE risk score and the TyG index was evaluated using Spearman's correlation analysis. Univariate and multivariate Cox regression analyses were used to identify whether the TyG index and GRACE risk score were independent predictors of the primary endpoint. C-statistics were calculated using receiver operating characteristic (ROC) analysis curves, which reflect the discrimination of the model. The areas under the curves (AUCs) of the two models were compared using the Delong test. The net reclassification improvement (NRI) and integrated differentiation improvement (IDI) risk models were used to compare the GRACE score model with the new risk model to identify which can correctly reclassify 2-year adverse cardiovascular disease. The clinical values of the new risk model and GRACE score alone were assessed using decision curve analysis (DCA). Data were analyzed using R (version 4.1.2; R Foundation for Statistical Computing, Vienna, Austria) and MedCalc (version 19.1; MedCalc Software Ltd., Ostend, Belgium). A two-sided *P*-value of <0.05 was considered to indicate statistical significance.

## Results

### Baseline characteristics of patients

This study included 515 patients with NSTE-ACS undergoing PCI, with a mean age of 62.3 ± 10.1 years and a male proportion of 70.1%. During the 24-month follow-up period, 56 patients (10.9% of the total population) experienced primary endpoint events, which consisted of 17 (3.3%) all-cause deaths, 15 (2.9%) non-fatal myocardial infarctions, 7 (1.4%) non-fatal ischemic strokes, and 17 (3.3%) ischemia-driven revascularizations. The baseline characteristics of the study population are shown in Table 1.

**Table 1:** Baseline characteristics of the study population.

	**ALL ** **(*n* = 515)**	**Without event** ** (*n* = 459)**	**With event** ** (*n* = 56)**	***P*-value**
Age (years)	62.3 ± 10.1	61.8 ± 10.0	66.3 ± 9.92	0.002
Male, *n* (%)	361 (70.1)	323 (70.4)	38 (67.9)	0.816
Obesity, *n* (%)	208 (40.4)	183 (39.9)	25 (44.6)	0.587
Systolic BP (mmHg)	131 ± 19.2	131 ± 19.5	130 ± 17.0	0.859
Diastolic BP(mmHg)	78.1 ± 13.0	78.1 ± 12.9	78.4 ± 13.5	0.894
Heart rate (bpm)	75.6 ± 12.3	75.4 ± 12.4	76.9 ± 11.9	0.379
Medical history and risk factors, *n* (%)
Hypertension	283 (55.0)	251 (54.7)	32 (57.1)	0.836
Diabetes	189 (36.7)	159 (34.6)	30 (53.6)	0.009
Previous or current smoking	163 (31.7)	150 (32.7)	13 (23.2)	0.199
Previous PCI	88 (17.1)	72 (15.7)	16 (28.6)	0.026
LVEF (%)	58.3 ± 7.74	58.7 ± 7.37	55.0 ± 9.75	0.009
GRACE score	119 ± 27.6	116 ± 24.2	151 ± 32.9	<0.001
TyG index	8.63 ± 0.47	8.59 ± 0.44	9.00 ± 0.49	<0.001
Laboratory values at hospital admission
WBC count ( ×109/L)	7.01 ± 2.70	6.99 ± 2.73	7.21 ± 2.47	0.539
Hemoglobin (g/L)	130 ± 17.4	130 ± 17.5	128 ± 16.3	0.336
Creatinine, μmol/L	80.0 ± 40.1	79.6 ± 40.6	84.0 ± 35.0	0.379
Uric acid (mmol/L)	317 ± 97.4	317 ± 96.8	321 ± 103	0.788
eGFR, mL/min	87.5 ± 20.9	88.0 ± 21.0	83.4 ± 19.4	0.114
FBG (mmol/L)	5.82 ± 1.57	5.70 ± 1.48	6.77 ± 1.92	<0.001
HbA1c (%)	6.36 ± 1.11	6.32 ± 1.07	6.67 ± 1.33	0.061
TC (mmol/L)	3.60 ± 0.97	3.59 ± 0.96	3.68 ± 1.07	0.552
TG (mmol/L)	1.36 ± 0.56	1.32 ± 0.52	1.70 ± 0.74	<0.001
HDL-C (mmol/L)	1.07 ± 0.30	1.07 ± 0.30	1.10 ± 0.26	0.356
LDL-C (mmol/L)	2.20 ± 0.82	2.18 ± 0.83	2.39 ± 0.76	0.052
Angiographic coronary anatomy, *n* (%)
One-vessel disease	93 (18.1)	83 (18.1)	10 (17.9)	1.000
Multi-vessel	270 (52.4)	228 (49.7)	42 (75.0)	0.001
Number of stents (*n*)	1.78 ± 1.32	1.78 ± 1.32	1.79 ± 1.28	0.956
Clinical diagnosis, *n* (%)				0.195
Unstable angina	249 (48.3)	227 (49.5)	22 (39.3)	
NSTEMI	266 (51.7)	232 (50.5)	34 (60.7)	
Medications at discharge, *n* (%)
Aspirin	514 (99.8)	458 (99.8)	56 (100)	-
Clopidogrel/Ticagrelor	515 (100)	459 (100)	56 (100)	-
Statin	503 (97.7)	449 (97.8)	54 (96.4)	0.629
ACEI/ARB/ARNI	353 (68.7)	310 (67.7)	43 (76.8)	0.210
β-blockers	438 (85.2)	387 (84.5)	51 (91.1)	0.270

PCI, percutaneous coronary intervention; LVDd, left ventricular end-diastolic dimension; LVEF, left ventricular ejection fraction; GRACE score, Global Registry of Acute Coronary Events score; TyG index, triglyceride-glucose index; WBC, white blood cell; eGFR, estimated glomerular filtration rate; FBG, fasting blood glucose; HbA1c, glycosylated hemoglobin A1c; TC, total cholesterol; TG, triglycerides; LDL-C, low-density lipoprotein cholesterol; HDL-C, high-density lipoprotein cholesterol; NSTEMI, non-ST-segment elevation myocardial infarction; UA, unstable angina; ACEI, angiotensin-converting enzyme inhibitor; ARB, angiotensin receptor blocker; ARNI, angiotensin receptor neprilysin inhibitor.

Patients were divided into two groups according to the occurrence of primary endpoint events. Patients with a primary endpoint event had a higher rate of smoking and prior histories of PCI and multivessel disease and a higher GRACE score, TyG index, age, FBG, glycated hemoglobin (HbA1c), TG, low-density lipoprotein cholesterol (LDL-C), and lower left ventricular ejection fraction (LVEF) than those without a primary endpoint event. We performed a correlation analysis and found a weak correlation between the TyG index and the GRACE score (*r* = 0.120, *P* = 0.006) (Supplementary Table 2).

### TyG index as an independent predictor of major endpoint events

**Figure 1:**
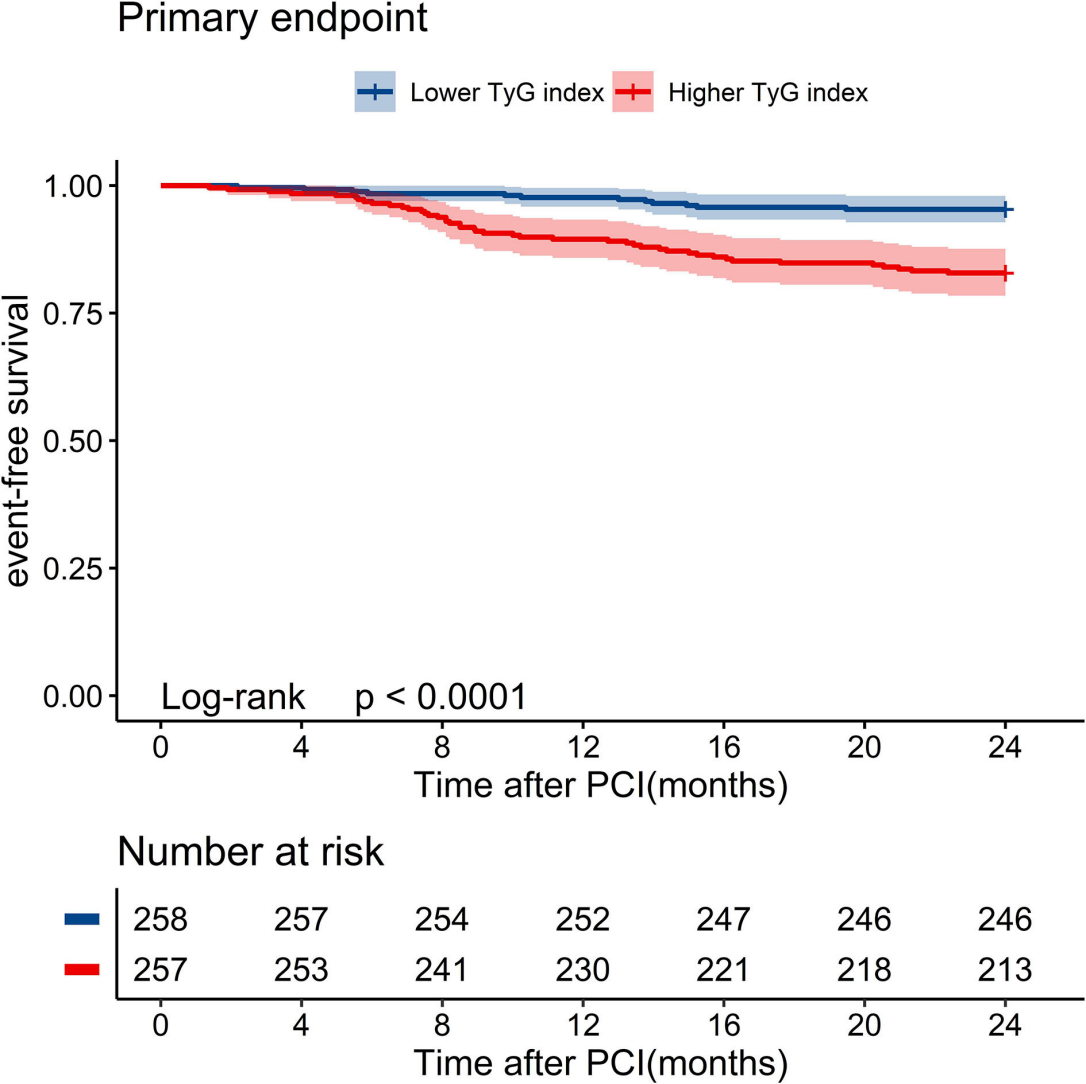
Kaplan–Meier survival curves of event-free survival stratified by the median level of TyG index for 2-year primary endpoint events. PCI, percutaneous coronary intervention; TyG index, triglyceride-glucose index.

**Figure 2:**
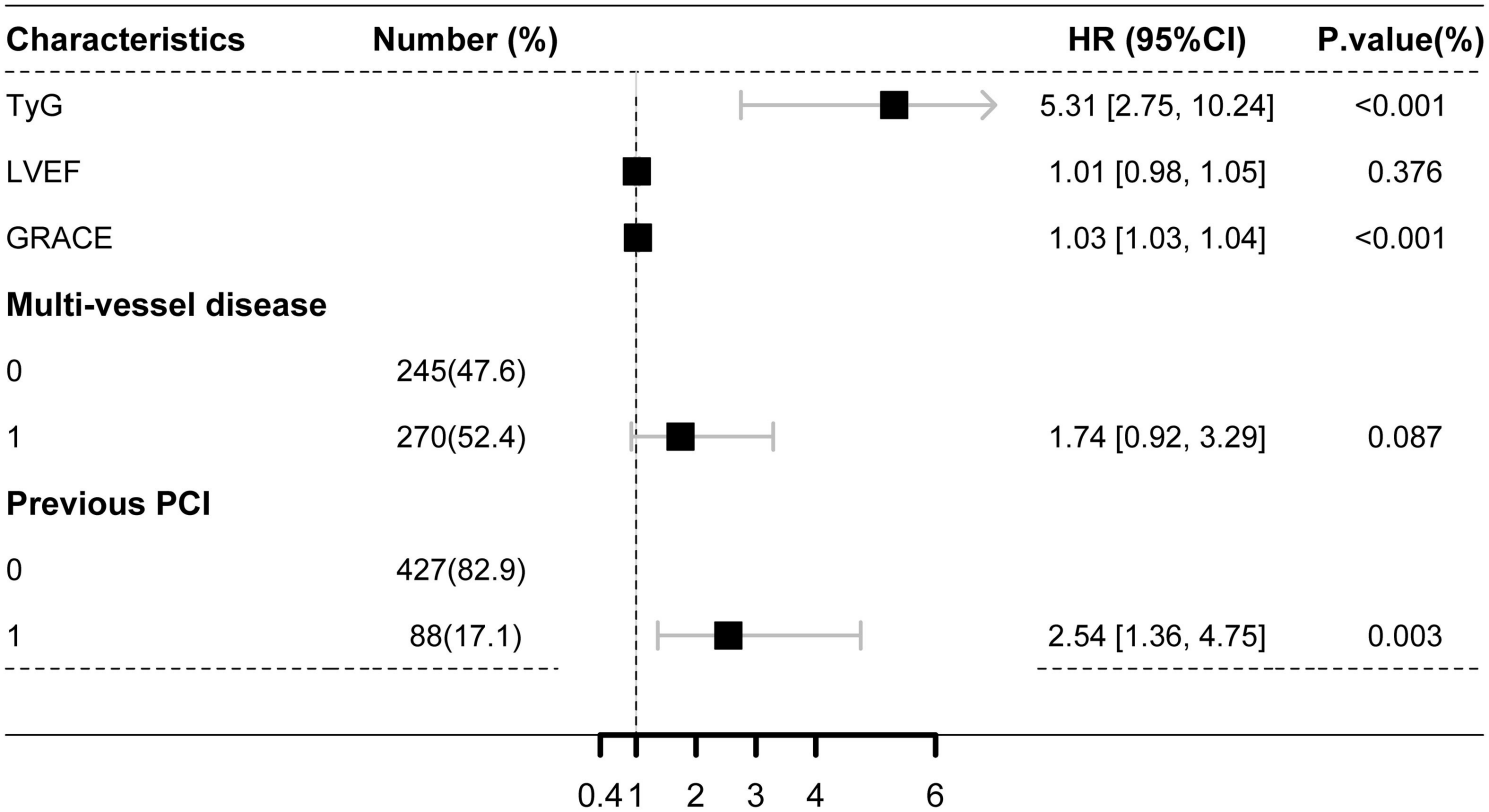
Multivariate cox analysis for the predictors of the primary endpoint. GRACE score, Global Registry of Acute Coronary Events score; TyG index, triglyceride-glucose index; LVEF, left ventricular ejection fraction; HR, hazard ratio; CI, confidence interval.

Kaplan-Meier curves stratified by the median TyG index are shown in Figure 1. Major endpoint events occurred more frequently in patients with a high TyG index (log-rank test, *P* < 0.001) (Figure 1). Univariate Cox analysis showed that the significant predictors of major endpoint events were smoking, multivessel disease, diabetes, history of PCI, lower LVEF, older age, higher HbA1C levels, higher LDL-C levels, higher FBG levels, higher TG levels, higher GRACE score, and higher TyG index (Supplementary Table 3). The results of the collinearity analysis of the TyG index and other predictors are displayed in Supplementary Table 4. TG, LDL-C, and HbA1C levels had high co-linearity with the TyG index. Therefore, the TG, LDL-C, and HbA1c levels were not included in the multivariate Cox analysis (Table 2). Multivariate Cox regression analysis revealed that the TyG index [hazard ratio (HR) 5.31; 95% confidence interval (CI) 2.75–10.24; *P* < 0.001] and GRACE score (HR 1.03; 95% CI 1.03–1.04; *P* < 0.001) were both independent predictors of major endpoints (Figure 2).

**Table 2:** Statistics for model improvement with the addition of the TyG index.

		***P*-value**
Events, *n* (%)	56 (10.9)	
Non-events, *n* (%)	459 (89.1)	
**Continuous NRI (%)**
cNRI _event_	43	
cNRI _non−event_	29	
cNRI	72 (95%CI 47–97)	<0.001
**IDI statistics**
IDI	0.086 (0.050–0.122)	<0.001
**AUC**
GRACE score	0.798 (95%CI 0.736–0.859)	
GRACE score + TyG	0.849 (95%CI 0.791–0.907)	
Difference	0.051	0.043

GRACE score, Global Registry of Acute Coronary Events score; TyG index, triglyceride-glucose index.

### Predictive value of combining TyG index and GRACE score for primary endpoint events

**Figure 3:**
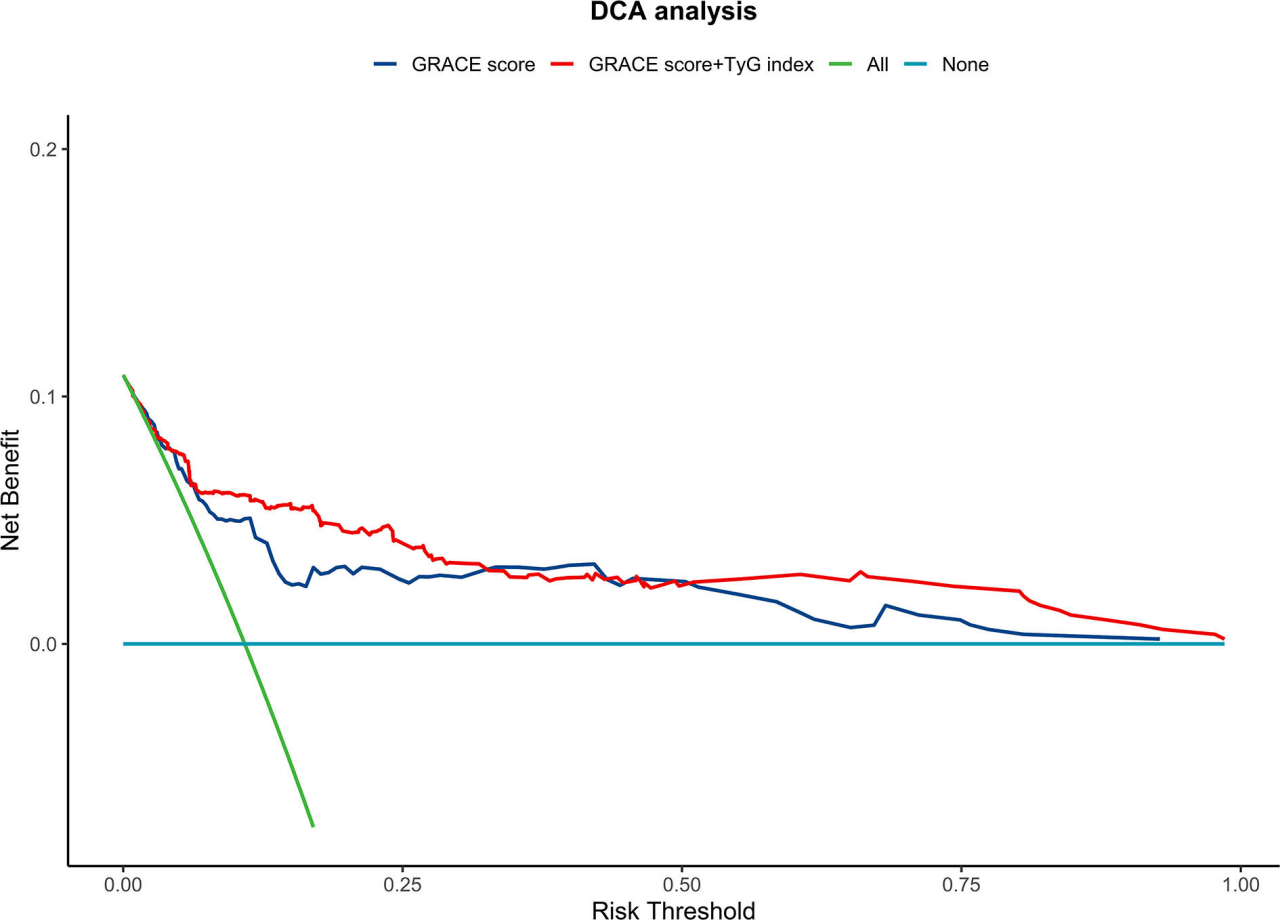
The decision curve analysis (DCA) comparing the GRACE risk score and its combination with the TyG index. GRACE score, Global Registry of Acute Coronary Events score; TyG index, triglyceride-glucose index.

In contrast with other parameters, adding the TyG index to the GRACE score resulted in the most significant improvement in the AUC for predicting primary endpoint events (AUCs: GRACE score 0.798 vs. +TyG index 0.849, *P* = 0.043; GRACE score 0.798 vs. +HbA1C 0.798, *P* = 0.959; GRACE score 0.798 vs. +FBG 0.810, *P* = 0.257; GRACE score 0.798 vs. +TG 0.841, *P* = 0.060; GRACE score 0.798 vs. +LDL-C 0.808, *P* = 0.254). Furthermore, adding the TyG index to the GRACE score resulted in a greater improvement in risk reclassification and discrimination (NRI = 0.718, *P* < 0.001; IDI = 0.086, *P* < 0.001) compared with HbA1C (NRI = 0.212, *P* = 0.127; IDI = 0.011, *P* = 0.123), FBG (NRI = 0.364, *P* = 0.010; IDI = 0.016, *P* = 0.070), TG (NRI = 0.591, *P* < 0.001; IDI = 0.052, *P* = 0.001), and LDL-C (NRI = 0.259, *P* = 0.066; IDI = 0.007, *P* = 0.216) (Tables 2, 3). DCA suggested that both the GRACE score and the new model (GRACE score+TyG index) had good clinical application value for predicting the 2-year primary endpoint. The net benefit of the new model was superior to the GRACE score alone, with a probability range of 0.04 to 0.32 (Figure 3).

**Table 3:** Prognostic predictive value of GRACE score when combined with other indexes.

	**ROC analysis**		**NRI**		**IDI**	
	**AUC (95% CI)**	***P*-value**	**Estimation (95% CI)**	***P*-value**	**Estimation (95% CI)**	***P*-value**
GRACE score	0.798 (0.736–0.859)	Reference		Reference		Reference
+HbA1C	0.798 (0.734–0.862)	0.959	0.212 (−0.061–0.485)	0.127	0.011 (−0.003–0.024)	0.123
+FBG	0.810 (0.748–0.872)	0.257	0.364 (−0.001–0.103)	0.010	0.016 (−0.001–0.033)	0.070
+TG	0.841 (0.789 – 0.894)	0.060	0.591 (0.322–0.861)	<0.001	0.052 (0.021–0.083)	0.001
+LDL–C	0.808 (0.747–0.868)	0.254	0.259 (−0.017–0.536)	0.066	0.007 (−0.004–0.018)	0.216
+TyG index	0.849 (0.791–0.907)	0.043	0.718 (0.466–0.971)	<0.001	0.086 (0.050–0.122)	<0.001

GRACE score, Global Registry of Acute Coronary Events score; TyG index, triglyceride-glucose index; FBG, fasting blood glucose; HbA1c, glycosylated hemoglobin A1c; TG, triglycerides; LDL-C, low-density lipoprotein cholesterol; IDI, integrated discrimination improvement; NRI, net reclassification improvement; CI, confidence interval.

## Discussion

In this study, we examined the prognostic and predictive effects of the GRACE score combined with the TyG index on 2-year adverse cardiovascular events in patients with NSTE-ACS who underwent elective PCI. The major findings of this study are as follows: (1) The GRACE score and TyG index could independently predict adverse cardiovascular events in patients with NSTE-ACS undergoing PCI; (2) the TyG index was weakly correlated with the GRACE score; and (3) in contrast to other glucose- and lipid-related parameters, adding the TyG index to the GRACE score showed the best performance in predicting adverse cardiovascular events.

Patients with IR are susceptible to a variety of metabolic disorders, such as hyperglycemia, dyslipidemia, and hypertension, which are closely related to a poor prognosis of CVD (cardiovascular disease) (11). The hyperinsulinemia-glucose clamp test and HOMA-IR are well-established methods for assessing IR but are difficult to implement in clinical practice because of their high cost, time requirement, and complexity (12). The TyG index is a newly discovered alternative marker for IR, which is simple, inexpensive, and convenient for clinicians. In addition, the TyG index shows a direct correlation with the hyperinsulinemia-glucose clamp test and HOMA-IR (3). At present, the TyG index is widely used in the assessment of CVD risk, and a clear correlation has been confirmed in various CVDs such as acute coronary syndrome, arteriosclerosis, heart failure, and coronary artery calcification (13–15).

However, the mechanisms responsible for the association between the TyG index and CVD remain uncertain. The potential relationship between the TyG index and CVD is as follows. First, IR can induce blood glucose and lipid metabolism disorders, leading to inflammation and oxidative stress, and these changes further aggravate the progression of CVD (16). Second, IR can induce increased production of glycosylated products and free radicals, resulting in NO inactivation and impaired vascular diastolic function (17). Moreover, IR can mediate the relevant signaling that sensitizes platelets to the anti-aggregation effects of prostaglandin I2 (PGI2) and NO, which, in turn, can lead to thrombosis (18). In addition, IR can induce excessive glycosylation, which promotes smooth muscle cell proliferation and collagen deposition, leading to increased ventricular stiffness, cardiac fibrosis, and ultimately, heart failure (11, 19). Finally, IR also plays an important role in hyperlipidemia. Increased TG levels can increase free fatty acid (FFA) levels and promote FFA flux from adipose tissue to non-adipose tissue (20). Furthermore, the retention of cholesterol- and TG-rich remnants of Apo B in coronary artery walls may play a role in the pathogenesis of atherosclerosis (21).

Owing to its ease of use, the convenience of data acquisition, and high accuracy, most of the current guidelines recommend the GRACE score to evaluate the prognosis of patients with NSTE-ACS. However, the biochemical indicators of the GRACE score include only myocardial necrosis markers and creatinine, leaving room for improvement in predicting adverse cardiovascular events. According to current research, pro-brain natriuretic peptides, C-reactive protein, and other biomarkers can contribute to the predictive power of the GRACE score (22, 23). The TyG index can reflect disorders of glucose and lipid metabolism and predict the prognosis of patients with ACS (4, 6, 24); however, the effect of the TyG index on the predictive ability of the GRACE scoring system for the occurrence of adverse cardiovascular events in patients with NSTE-ACS undergoing PCI remains unknown. Among our patients, we found that the GRACE score increased with an increasing TyG index, and a correlation was found between the two variables. Similar to previous studies, we found that an elevated TyG index and GRACE score were significantly associated with 2-year adverse cardiovascular events in patients with NSTE-ACS who underwent PCI. We found that the predictive ability can be significantly improved by including the TyG index in the GRACE score since the AUC went from 0.798 to 0.845 when the TyG index was combined with the GRACE score. Moreover, multiple novel methods were used to evaluate the improvement in discrimination and clinical usage of the TyG index when added to the GRACE score, including the IDI, NRI, and DCA curves. Using a continuous, category-free NRI, 29% of patients without events were reclassified as low risk and 43% of patients with events were reclassified as high risk. Furthermore, IDI analysis showed that incorporation of the TyG index with the GRACE scores significantly improved the discriminatory accuracy for adverse cardiovascular events with an IDI of 8.6% (*p* < 0.001). Finally, by plotting DCA curves, we found that the predictive value of the new model with the combined TyG index was higher than that of the traditional GRACE model, with a probability range of 0.04 to 0.32 for endpoint events. Therefore, with the assistance of this new model (GRACE score combined with TyG index), major endpoint events can be predicted more accurately and better clinical decisions can be made for patients with NSTE-ACS.

We then further compared the gain effect of the Tyg index and the levels of HbA1C, FBG, TG, and LDL-C on the GRACE score for predicting adverse cardiovascular events, considering that the TyG index is closely associated with lipid and glucose metabolism. We found that the TyG index was strongly correlated with TG levels and moderately correlated with FBG, TG, LDL-C, and HbA1C levels. Given the relatively small number of patients with diabetes in our study cohort and the small range of glycemic fluctuations, the TyG index and TG levels correlated more strongly. ROC curve analyses, NRI, and IDI were used to quantify the improvement in the different prediction models. Although previous studies have shown that HbA1C is an independent risk factor for cardiovascular events (25, 26), HbA1C did not significantly improve the predictive power of the GRACE score in this study. Since HbA1C reflects blood glucose levels over the past 3 months, and most hospitalized patients in this study did not have diabetes, there was less difference in HbA1C values. Patients with the acute coronary syndrome often have stress hyperglycemia. Acute elevated blood glucose levels can induce oxidative stress, leading to direct injury of myocardial cells, endothelial dysfunction, and activation of clotting pathways, which can lead to enlarged infarct size, restenosis, and adverse cardiac remodeling (27–30). Numerous studies have shown a strong association between hyperglycemia and poor outcomes in patients with ACS (31–33). In this study, fasting glucose improved the ability of the GRACE score to distinguish patients at high risk of major adverse cardiovascular events (MACE) (NRI: *P* = 0.010). In previous studies, low-density lipoprotein was a very important indicator of the prognosis of patients with AMI (34), but this study showed a limited additional effect on the GRACE score. This may be because patients had been treated with statins before hospitalization for CHD-related symptoms with or without PCI. Surprisingly, in this study, triglycerides played a significant role in enhancing the ability of the GRACE score to predict MACE. Studies have shown that in addition to LDL, hypertriglyceridemia plays an important role in the development of coronary artery disease (35–38). In a large cohort study by Ambrosy et al. elevated triglyceride levels were associated with an increased risk of atherosclerotic cardiovascular disease events and a reduced risk of death in a cohort treated with statins (39). Hypertriglyceridemia is closely related to patients' eating habits (40), so a healthy diet after discharge is essential. The TyG index reflects the status of insulin resistance and has a good predictive effect on the occurrence of MACE. Including these parameters into the GRACE score system, we found that the TyG index had a greater incremental impact on risk prediction and stratification than the other parameters. Thus, the TyG index may provide more information on recurrent adverse cardiovascular events.

Patients with multivessel coronary artery disease have a high risk of MACE (41). We found that patients with NSTE-ACS and a high TyG index were more likely to have the multivessel disease. Similar to our research, a recent study found that a high TyG was significantly associated with the risk of multivessel coronary artery disease regardless of confounders, such as age, sex, SBP, DBP, history of smoking and alcohol consumption, and drug therapy (42). This may be another reason the TyG index can predict primary endpoints independent of the GRACE score in some patients. Unfortunately, there are few studies on the relationship between TyG and multivessel coronary artery disease at present, and further large-sample studies are needed.

### Limitations

Our study has three limitations. First, it was a single-center retrospective study; therefore, the findings should be interpreted with caution. Second, because patient information was insufficient, we did not specify the cardiovascular deaths that were commonly used as endpoint events. Finally, as the study involved only Chinese patients, generalizing the findings to other ethnic groups might not be appropriate.

## Conclusion

Combining the TyG index and GRACE score could improve the prediction of the occurrence of 2-year adverse cardiovascular events. This new risk model could identify patients with NSTE-ACS at higher risk of adverse events following PCI so that they can be monitored more carefully.

## Data availability statement

The original contributions presented in the study are included in the article/Supplementary material, further inquiries can be directed to the corresponding author/s.

## Ethics statement

The studies involving human participants were reviewed and approved by the First Affiliated Hospital of Zhengzhou University. Written informed consent for participation was not required for this study in accordance with the national legislation and the institutional requirements.

## Author contributions

SP, GM, and XZ contributed to the conception and design of the study. SP, GM, YZ, and ZR collected the clinical information. SP wrote the first draft of the manuscript. SP, GM, and YD wrote sections of the manuscript. All authors have revised, read, and approved the final version of the manuscript.

## Funding

This study was supported by the Science and Technology Development of Henan Province (Grant No. 212102310210).

## Conflict of interest

The authors declare that the research was conducted in the absence of any commercial or financial relationships that could be construed as a potential conflict of interest.

## Publisher's note

All claims expressed in this article are solely those of the authors and do not necessarily represent those of their affiliated organizations, or those of the publisher, the editors and the reviewers. Any product that may be evaluated in this article, or claim that may be made by its manufacturer, is not guaranteed or endorsed by the publisher.

## Abbreviations

AUC: area under the curve
CI: confidence interval
DCA: decision curve analysis
FBG: fasting blood glucose
GRACE: Global Registry of Acute Coronary Events
HbA1c: glycated hemoglobin
HOMA-IR: homeostatic model assessment for insulin resistance
HR: hazard ratio
IDI: integrated differentiation improvement
IR: insulin resistance
LDL-C: low-density lipoprotein cholesterol
LVEF: left ventricular ejection fraction
NRI: net reclassification improvement
NSTE-ACS: non-ST-segment elevation acute coronary syndrome
NSTEMI: non-ST-segment elevation myocardial infarction
PCI: percutaneous coronary intervention
ROC: receiver operating characteristic
TG: triglycerides
TyG index: triglyceride-glucose index
UA: unstable angina.
