# Corrigendum: Addition of TyG index to the GRACE score improves prediction of adverse cardiovascular outcomes in patients with non-ST-segment elevation acute coronary syndrome undergoing percutaneous coronary intervention: A retrospective study

**DOI:** 10.3389/fcvm.2022.1037968

**Published:** 2022-09-22

**Authors:** Shuo Pang, Guangrui Miao, Yuanhang Zhou, Yang Du, Ziao Rui, Xiaoyan Zhao

**Affiliations:** Department of Cardiology, The First Affiliated Hospital of Zhengzhou University, Zhengzhou, China

**Keywords:** acute coronary syndrome, percutaneous coronary intervention, insulin resistance, biomarkers, prognosis

In the published article, there was an error in the Funding statement [The funding statement for the Science and Technology Development of Henan Province was displayed as “202102310210.” The correct statement is “Science and Technology Development of Henan Province (212102310210).”]. The correct Funding statement appears below.

## Funding

This study was supported by the Science and Technology Development of Henan Province (Grant No. 212102310210).

The authors apologize for this error and state that this does not change the scientific conclusions of the article in any way. The original article has been updated.

## Publisher's note

All claims expressed in this article are solely those of the authors and do not necessarily represent those of their affiliated organizations, or those of the publisher, the editors and the reviewers. Any product that may be evaluated in this article, or claim that may be made by its manufacturer, is not guaranteed or endorsed by the publisher.

