# Supplementary material for: Addition of TyG index to the GRACE score improves prediction of adverse cardiovascular outcomes in patients with non-ST-segment elevation acute coronary syndrome undergoing percutaneous coronary intervention: A retrospective study
Source: Front Cardiovasc Med. 2022 Aug 25;9:957626. doi: 10.3389/fcvm.2022.957626 (PMC9453480; doi:10.3389/fcvm.2022.957626)
Supplement: Supplementary file 1 [file Table_1.DOCX]

Supplementary Material

**Supplementary Figures and Tables**


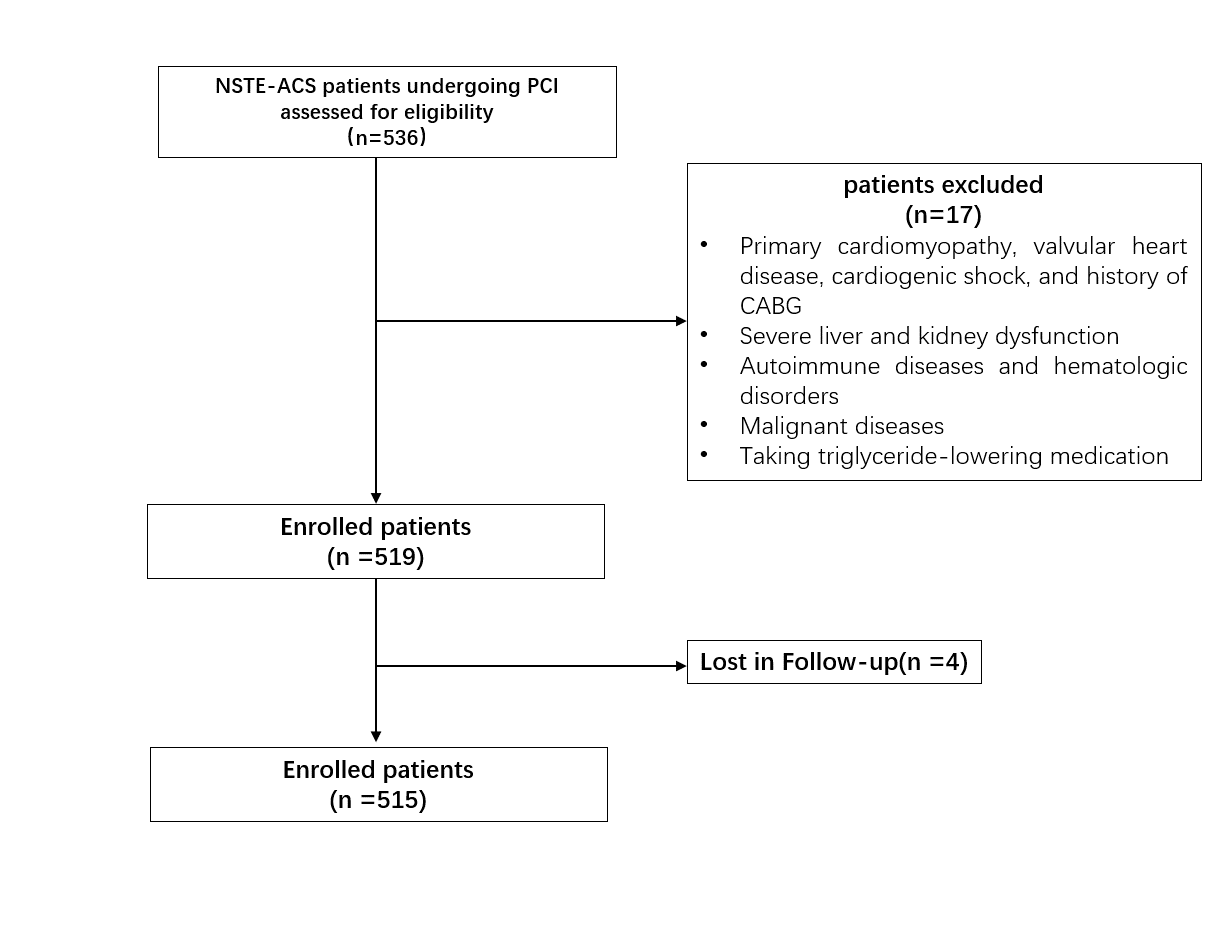


**Supplementary Figure 1.** **Study protocol flow chart**

Abbreviations: PCI, percutaneous coronary intervention; CABG，Coronary artery bypass grafting

**Supplemental Table 1. GRACE score for NSTE-ACS**

| Age | Pts | Heart Rate (bpm) | Pts | Systolic BP (mmHg) | Pts | Creatinine Level (mg/dL) | Pts | Killip classification | Pts | Risk factors | Pts |
| --- | --- | --- | --- | --- | --- | --- | --- | --- | --- | --- | --- |
| <30 | 0 | <50 | 0 | <80 | 58 | 0 - 0.39 | 1 | Ⅰ | 0 | Cardiac arrest at admission | 39 |
| 30 - 39 | 8 | 50 - 69 | 3 | 80 - 99 | 53 | 0.40 - 0.79 | 4 | Ⅱ | 29 | ST segment deviation | 28 |
| 40 - 49 | 25 | 70 - 89 | 9 | 100 - 119 | 43 | 0.8 - 1.19 | 7 | Ⅲ | 39 | Abnormal cardiac enzymes | 14 |
| 50 - 59 | 41 | 90 - 109 | 15 | 120 - 139 | 34 | 1.20 - 1.59 | 10 | Ⅳ | 59 |  |  |
| 60 - 69 | 58 | 110 - 149 | 24 | 140 - 159 | 24 | 1.6 - 1.99 | 13 |  |  |  |  |
| 70 - 79 | 75 | 150 - 199 | 38 | 160 - 199 | 10 | 2.0 - 3.99 | 21 |  |  |  |  |
| 80 - 89 | 91 | ≥200 | 46 | ≥200 | 0 | ≥4 | 28 |  |  |  |  |

**Supplemental Table 2. Correlations between  the  TyG index and cardiovascular risk factors.**

|  | **Correlation** | **P value** |
| --- | --- | --- |
| age | -0.06 | 0.183 |
| GRACE | 0.12 | 0.006 |
| LVEF | -0.09 | 0.041 |
| diabete1 | 0.26 | ＜0.001 |
| FBG | 0.46 | ＜0.001 |
| HbA1C | 0.30 | ＜0.001 |
| TG | 0.82 | ＜0.001 |
| LDL-C | 0.30 | ＜0.001 |
| Multi vessel disease | 0.17 | ＜0.001 |
| Previous PCI | -0.12 | 0.006 |

Abbreviations: PCI, percutaneous coronary intervention; LVEF, left ventricular ejection fraction; GRACE score, Global Registry of Acute Coronary Events score; TyG index, triglyceride-glucose index; HbA1c, glycosylated hemoglobin A1c; LDL-C, low-density lipoprotein cholesterol.

**Supplemental Table 3. Univariate Cox proportional hazards analysis for the primary endpoint**

|  | Univariate analysis | |  |
| --- | --- | --- | --- |
|  | HR | 95% CI | P value |
| Age (per 1 year) | 1.044 | 1.016-1.072 | 0.002 |
| Male (vs. female) | 0.908 | 0.518-1.59 | 0.735 |
| Obesity | 1.188 | 0.701-2.012 | 0.522 |
| SBP (per 1 mmHg) | 0.999 | 0.985-1.012 | 0.857 |
| DBP (per 1 mmHg) | 1.001 | 0.981-1.022 | 0.909 |
| Heart rate (per 1 bpm) | 1.009 | 0.989-1.029 | 0.372 |
| Hypertension | 1.101 | 0.649-1.870 | 0.721 |
| Diabetes | 2.049 | 1.212-3.465 | 0.007 |
| Previous or current Smoking | 0.642 | 0.345-1.194 | 0.162 |
| Previous PCI | 2.054 | 1.15-3.668 | 0.015 |
| LVEF (per 1 %) | 0.956 | 0.932-0.982 | 0.001 |
| WBC count (per 10^9^/L) | 1.026 | 0.942-1.116 | 0.560 |
| Hemoglobin (per 1 g/L) | 0.993 | 0.979-1.008 | 0.344 |
| Creatine (per 1 µmol/L) | 1.002 | 0.997-1.007 | 0.404 |
| Uric acid (per 1 mmol/L) | 1.000 | 0.998-1.003 | 0.738 |
| eGFR (per 1 mL/ (min × 1.73 m^2^) | 0.991 | 0.979-1.003 | 0.123 |
| GRACE score | 1.041 | 1.032-1.05 | <0.001 |
| TyG index | 9.421 | 4.827-18.387 | <0.001 |
| FBG (per 1 mmol/L) | 1.284 | 1.145-1.439 | <0.001 |
| HbA1c (per 1 %) | 1.249 | 1.029-1.516 | 0.024 |
| TC (per 1 mmol/L) | 1.083 | 0.828-1.415 | 0.561 |
| TG (per 1 mmol/L) | 2.542 | 1.782-3.627 | <0.001 |
| HDL-C (per 1 mmol/L) | 1.418 | 0.607-3.312 | 0.420 |
| LDL-C (per 1 mmol/L) | 1.306 | 0.97-1.759 | 0.078 |
| One-vessel disease | 0.986 | 0.497-1.953 | 0.967 |
| Multi-vessel disease | 2.840 | 1.551-5.2 | 0.001 |
| Number of stents (per 1 stent) | 1.001 | 0.821-1.22 | 0.992 |
| NSTEMI (vs. Unstable angina) | 1.472 | 0.861-2.516 | 0.158 |
| Statin at discharge | 0.631 | 0.154-2.586 | 0.522 |
| ACEI/ARB/ARNI at discharge | 1.552 | 0.835-2.882 | 0.165 |

Abbreviations: PCI, percutaneous coronary intervention; **LVDd**, Left ventricular end diastolic dimension; LVEF, left ventricular ejection fraction; GRACE score, Global Registry of Acute Coronary Events score; TyG index, triglyceride-glucose index; WBC, white blood cell; eGFR, estimated glomerular filtration rate; FBG, fasting blood glucose; HbA1c, glycosylated hemoglobin A1c; TC, total cholesterol; TG, triglycerides; LDL-C, low-density lipoprotein cholesterol; HDL-C, high-density lipoprotein cholesterol; ACEI, angiotensin converting enzyme inhibitor; ARB, angiotensin receptor blocker; ARNI, angiotensin receptor neprilysin inhibitor.

**Supplemental Table 4. Co-linearity analysis of the primary endpoint predictors and TyG index.**

|  | Unstandardized coefficients | | Standardized coefficients  Beta | t | Sig. | Collinearity statistics | |
| --- | --- | --- | --- | --- | --- | --- | --- |
|  | B | Std. error |  |  |  | Tolerance | VIF |
| (Constant) | 7.809 | 0.245 |  | 31.832 | 0.000 |  |  |
| Age, year | -0.008 | 0.002 | -0.172 | -3.402 | 0.001 | 0.589 | 1.697 |
| Multi vessel disease | 0.093 | 0.037 | 0.100 | 2.511 | 0.012 | 0.949 | 1.053 |
| Diabetes | 0.099 | 0.052 | 0.102 | 1.897 | 0.058 | 0.514 | 1.946 |
| Previous PCI | -0.110 | 0.048 | -0.088 | -2.263 | 0.024 | 0.985 | 1.016 |
| HbA1C, % | 0.099 | 0.023 | 0.234 | 4.361 | 0.000 | 0.520 | 1.925 |
| LDL-C, mmol/L | 0.161 | 0.023 | 0.283 | 7.120 | 0.000 | 0.951 | 1.051 |
| GRACE score | 0.003 | 0.001 | 0.172 | 3.267 | 0.001 | 0.539 | 1.856 |
| LVEF | -0.001 | 0.003 | -0.023 | -0.545 | 0.586 | 0.861 | 1.162 |

Abbreviations: PCI, percutaneous coronary intervention; LVEF, left ventricular ejection fraction; GRACE score, Global Registry of Acute Coronary Events score; TyG index, triglyceride-glucose index; HbA1c, glycosylated hemoglobin A1c; LDL-C, low-density lipoprotein cholesterol.
